# Genome-Wide Fitness and Expression Profiling Implicate Mga2 in Adaptation to Hydrogen Peroxide

**DOI:** 10.1371/journal.pgen.1000488

**Published:** 2009-05-29

**Authors:** Ryan Kelley, Trey Ideker

**Affiliations:** 1 Program in Bioinformatics, University of California San Diego, La Jolla, California, United States of America; 2 Department of Bioengineering, University of California San Diego, La Jolla, California, United States of America; The University of Queensland, Australia

## Abstract

Caloric restriction extends lifespan, an effect once thought to involve attenuation of reactive oxygen species (ROS) generated by aerobic metabolism. However, recent evidence suggests that caloric restriction may in fact raise ROS levels, which in turn provides protection from acute doses of oxidant through a process called adaptation. To shed light on the molecular mechanisms of adaptation, we designed a series of genome-wide deletion fitness and mRNA expression screens to identify genes involved in adaptation to hydrogen peroxide. Combined with known transcriptional interactions, the integrated data implicate Yap1 and Skn7 as central transcription factors of both the adaptive and acute oxidative responses. They also identify the transcription factors Mga2 and Rox1 as active exclusively in the adaptive response and show that Mga2 is essential for adaptation. These findings are striking because Mga2 and Rox1 have been thought to control the response to hypoxic, not oxidative, conditions. Expression profiling of *mga2Δ* and *rox1Δ* knockouts shows that these factors most strongly regulate targets in ergosterol, fatty-acid, and zinc metabolic pathways. Direct quantitation of ergosterol reveals that its basal concentration indeed depends on Mga2, but that Mga2 is not required for the decrease in ergosterol observed during adaptation.

## Author Summary

Reactive oxygen species (ROS) damage a variety of structures within the cell, resulting in disease and aging. In a seemingly paradoxical effect termed adaptation, it is possible to prevent damage caused by ROS by pre-treating the cell with a small amount of oxidant. We studied this process in order to identify the mechanisms that provide this protection. Our study identified a number of genes and processes with previously unappreciated roles in adaptation. The mechanisms we identified are remarkable because they are distinct from those previously known to protect the cell from ROS. Although this study is conducted in yeast, the wide conservation of adaptation among many organisms suggests that the results from this study may be widely applicable.

## Introduction

Oxidative stress is caused by a number of reactive oxygen species (ROS) generated as a result of aerobic metabolism or chemical exposure. These compounds damage a variety of cellular products, including DNA, proteins, and lipid membranes, and are associated with a number of human pathologies. For example, in cardiovascular disease, oxidation of low-density lipoprotein causes an inflammatory response [1]. The sensitivity of neurons to oxidative stress implicates ROS in neurodegenerative diseases, such as Parkinson's and Alzheimer's [2]–[4].

A continuing source of controversy is the role of oxidative stress in aging. Caloric restriction has been shown to extend lifespan in a number of species [5]. Initially, it was hypothesized that the effect on lifespan occurs primarily because caloric restriction reduces the level of aerobic respiration, a major source of ROS [6]. Newer evidence is challenging this hypothesis, since caloric restriction paradoxically increases respiration [7]. Increased respiration, in turn, can generate mild levels of ROS which protect against high doses of oxidant [8]. This process is known as adaptation or hormesis [9] and is widely conserved among eukaryotes [8], [10]–[12]. One hypothesis is that adaptation to oxidative stress is the basis for the lifespan-extending effect of caloric restriction [13],[14]. Thus, further efforts to understand the process of adaptation may have broad implications on models of aging and disease.

In one model of adaptation, the cell increases the activity of the enzymes and pathways required to rid the cells of ROS, leaving it better equipped to process acute dosages of oxidant when they arise. Under this model, genes involved in the adaptive response are expected to be a subset of those that become active in the acute response [15]. Many such candidates have been identified, including a variety of biosynthetic enzymes which produce small molecular compounds or proteins with reduction potential, such as glutathione (GSH), thioredoxin, NADPH, and trehalose [16]–[20]. Different enzymes facilitate this process for different ROS, including catalases and peroxidases (which deal with peroxide radicals) [21],[22] and superoxide dismutases (which deal with superoxide radicals) [23],[24]. Additional proteins serve to repair the damage caused by oxidative stress. Heat shock proteins act as chaperones within the cell, allowing damaged proteins to fold properly or preparing them for disposal [25]. DNA repair genes are also vital, as oxidative stress can damage both nucleotides and the phosphodiester DNA backbone [26]. Several studies have implicated classical oxidative stress proteins and pathways in adaptation, including the transcription factor Yap1 [27] and glutathione synthesis [28]–[30].

In contrast to this model, a second body of evidence suggests that adaptation may be governed by novel pathways not directly involved in the response to acute oxidation. In a study of adaptation to the oxidant linoleic acid, Alic *et al.* found that adaptation can occur without induction of oxidative or general stress response genes following pretreatment [31]. Instead, various metabolic processes were activated and protein synthesis was inhibited. Moreover, machinery with a central role in the acute response, such as the mitochondria [9],[32] or the Msn2/4 environmental stress response factors, are not required for adaptation [27],[33].

Nonetheless, expression studies of acute oxidative damage have helped to identify a set of genes involved in the common environmental stress response (ESR) and implicated the Msn2/4 transcription factors in control of this gene set [34]–[36]. In fitness studies of yeast deletion strains, Thorpe *et al.* identified a set of genes required for the response to hydrogen peroxide, mainly dealing with the proper functioning of the mitochondria [37]. However, to-date these genome-scale approaches have focused on the acute, rather than the adaptive, response. One study to date that has screened for adaptive genes focused on a set of 268 genes selected based on previous literature [38].

Here, we use the rich functional genomics toolbox of yeast to identify pathways involved in adaptation to hydrogen peroxide. To accomplish this goal, we use barcode arrays to screen the *Saccharomyces cerevisiae* gene deletion collection [39] for genes required in the acute and adaptive responses, and we couple these data with genome-wide mRNA expression profiles to build a system-wide model of adaptation.

## Results/Discussion

### A Genetic Screen to Identify Genes Functioning in Adaptation

**Figure 1:**
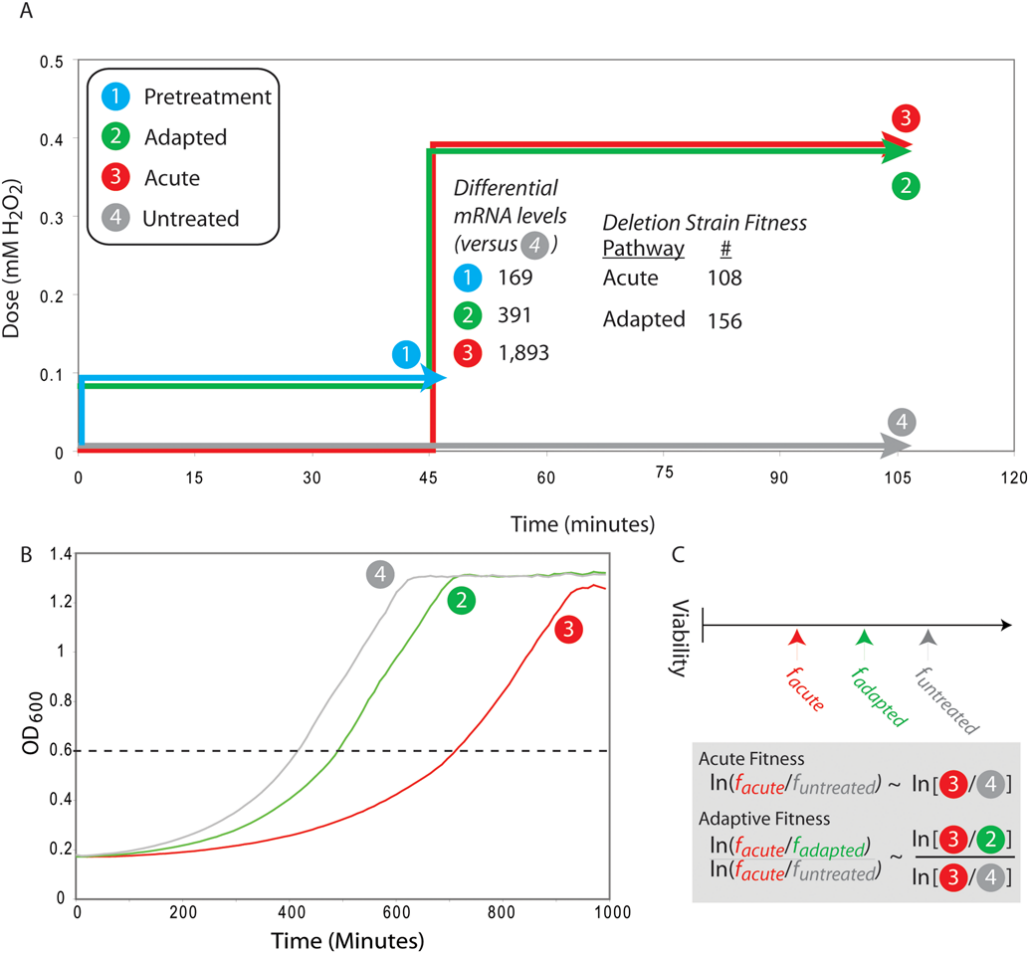
Study design. A. Yeast cells were collected following each of four hydrogen peroxide treatment conditions (pretreated, adapted, acute, and untreated, labeled 1–4). Competitive growth experiments were performed between gene deletion pools grown in adapted versus acute conditions (to identify genes required specifically for adaptation) and between pools grown in acute versus untreated conditions (to identify genes required for the acute response). Gene expression profiling was performed in either adapted or acute conditions versus untreated cells. B. Profiling of *wild type* growth reveals that pretreatment with mild hydrogen peroxide (green) leads to improved recovery to an OD_600_ threshold (dashed line) compared to no pretreatment (red) following a high dose of hydrogen peroxide. An enlarged version of panel B is available as Figure S1. C. For an individual gene deletion, the acute sensitivity is defined as the difference between the acute and untreated viability. The adapted sensitivity is the fraction of that difference that is recovered by mild pretreatment with hydrogen peroxide.

As shown in Figure 1A, we elicited adaptation using a protocol consisting of a mild pretreatment of hydrogen peroxide (0.1 mM H_2_O_2_ for 45 min) followed by a later high dose (0.4 mM H_2_O_2_ for 1 hr). For purposes of comparison, we also conducted an acute protocol which exposed cells to the high dose only (0.4 mM H_2_O_2_ for 1 hr). Consistent with previous findings [9], we observed that yeast cells undergoing the adaptation protocol exhibited a smaller reduction in viability compared to cells exposed to the acute treatment protocol (Figure 1B and Figure S1).

Given these protocols, we designed a series of yeast genome-wide phenotyping experiments using the publicly available pool of 4,831 viable single-gene deletion strains [40]. Each strain in the pool incorporates a pair of unique oligonucleotide barcode tags, which allow the relative prevalence of all strains to be tracked in growth experiments by hybridization of pooled genomic DNA to a barcode microarray. In a first experiment, two identical pools of deletion mutants were treated with the adaptation or acute protocol, respectively, and directly compared on a barcode array (with multiple biological replicates; see Methods). In a second experiment, a pool subjected to the acute treatment was compared against an untreated pool.

These experiments were used to identify genes required for adaptation or for the acute response, as shown in Figure 1C. Fitness in the acute response was defined as the difference in viability between the acute and untreated conditions (determined from the log ratio of intensities measured in the direct comparison of the acute and untreated pools, see Methods). Adaptive fitness was defined as the difference in viability between the acute and adapted conditions, normalized by the magnitude of the acute effect (Figure 1C).

### Genes Required for Adaptation Do Not Function in Canonical Oxidative Stress Pathways

**Figure 2:**
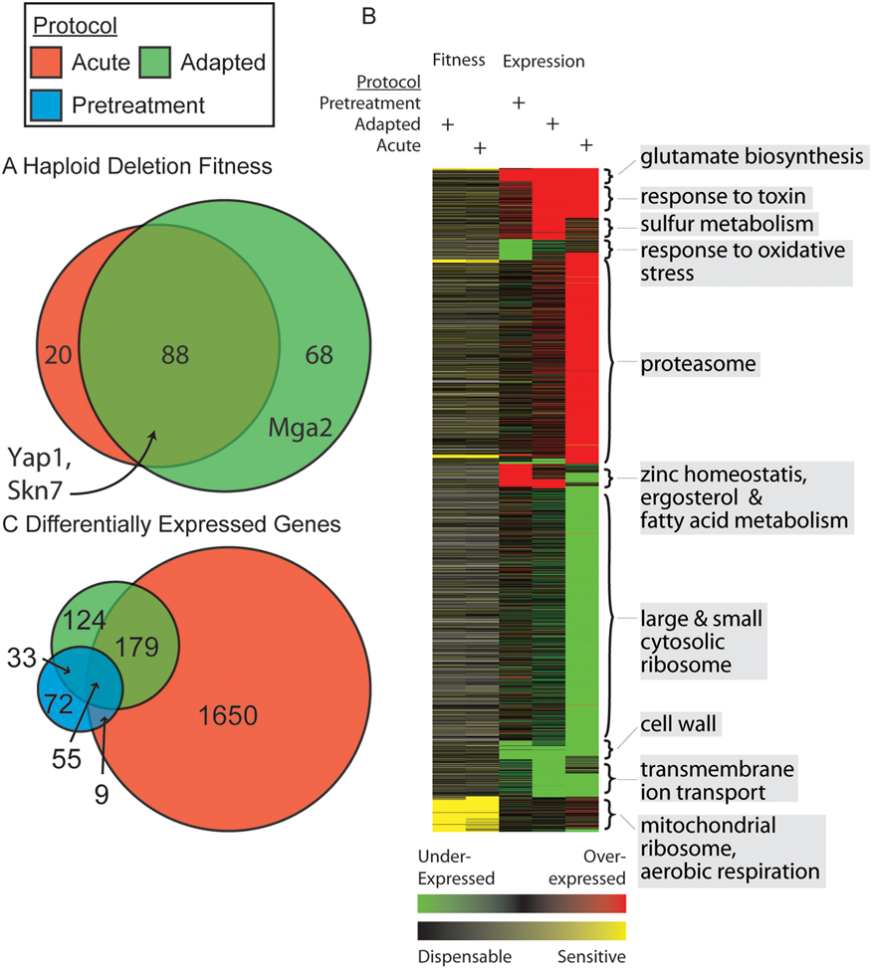
Fitness and expression profiling overview. A. Numbers and overlap of gene deletions that are sensitive in the adaptive (green) and acute (red) treatment protocols. B. Hierarchical clustering of the differentially expressed or sensitive genes from each screen. Clusters are annotated at right with over-represented functional groups. C. Numbers and overlap of differentially expressed genes identified in each of the three expression treatment protocols.

A total of 156 versus 108 genes were found to be required for the adaptive versus the acute responses, with an overlap of 88 genes (Figure 2A). A complete list of acute and adaptation-sensitive genes is provided in the Dataset S1. Surprisingly, neither the adaptive nor the acute screen was enriched for oxidative stress response genes (GO Biological Process 0006979) which encode enzymes involved in processes such as ROS detoxification and homeostasis. This may be due to the ability of this response to compensate for the loss of single gene activities, confirming earlier observations regarding the acute response by Thorpe *et al.* (Table S1) [37]. Instead, both the adaptive and acute gene sets were heavily enriched for functions in the mitochondrial ribosome and aerobic respiration (Figure 2B). The identification of these functions is puzzling in light of an earlier finding that yeast with defective mitochondria (rho^−^ mutants) adapt to oxidative stress [9],[32]. In these studies, a milder high dose was required to demonstrate adaptation; therefore, the observed deficiency in adaptation of mitochondrial mutants in our screen may be due to increased sensitivity to the high dose.

### Adaptation Requires Transcriptional Regulators

Both sensitivity screens also highlighted several transcription factors (Figure 2A), which are particularly interesting due to their potential roles in regulation of adaptation. These factors include *YAP1* and *SKN7* which, in contrast to the above enrichment results, do have known involvement in the response to oxidative stress [41],[42]. *YAP1* and *SKN7* were previously identified as adaptive-sensitive in the restricted screen conducted by Ng *et al.* [38]. The transcription factor *MGA2* was required for the adaptive but not the acute response. *MGA2* has been implicated in fatty-acid biosynthesis and the response to hypoxia [43].

**Figure 3:**
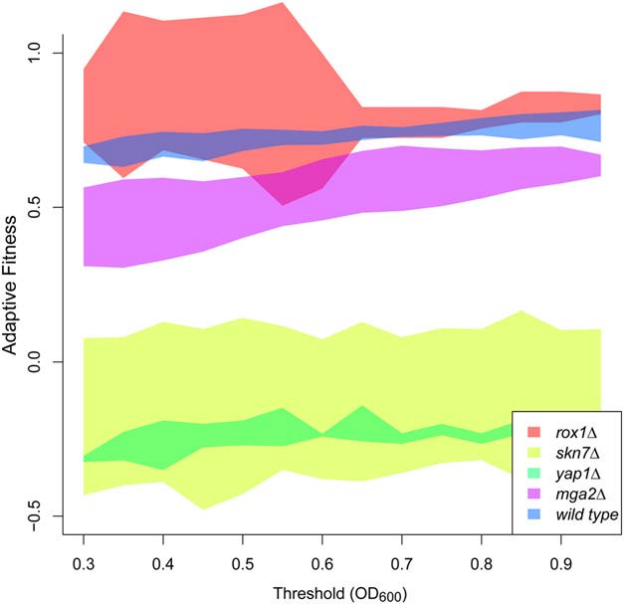
Confirmation of mutant strains deficient in adaptation. Growth curves in untreated, adapted, and acute oxidative conditions were measured for *wild type* and each of four deletion strains starting from single-cell colonies. These curves were used to compute adaptive fitness (y-axis) which is shown over a range of OD_600_ threshold values (x-axis, see Methods). For all thresholds, an adaptation defect compared to *wild type* was confirmed for *yap1Δ*, *skn7Δ*, *mga2Δ* (all p<5.0×10^−2^ by unpaired t-test). No defect was observed for *rox1Δ*, which was also consistent with the genome-wide screen. Each colored band represents the range of adaptive fitness values spanned by the mean±2*standard error of multiple biological replicates.

To confirm the requirement of these transcription factors for oxidative adaptation, we performed additional adaptation experiments specifically in *yap1Δ*, *skn7Δ*, *mga2Δ*, and *wild type* strains. For each, we quantified the severity of each protocol (acute, adapted, untreated) as the time required to recover to a specific OD_600_ threshold following treatment (Figure 1B) [32]. Adaptive fitness was calculated as the reduction in viability of the adapted culture, relative to that of the acute-treated culture (see Methods). Figure 3 displays the computed fitness values for each strain over a range of OD_600_ thresholds. All of these strains were indeed confirmed to have fitness values less than *wild type*.

### Distinct Sets of Genes Are Expressed during the Adaptive versus Acute Responses

Next, we performed mRNA expression profiling on each of the three treatment protocols (pretreated, adapted, acute, see Figure 1A) in comparison to untreated conditions. These profiles were analyzed to identify two types of adaptive response genes: early versus late. Early adaptive genes were defined as those that were differentially expressed after the 45 min. pretreatment relative to untreated conditions (169 genes at p<1.0×10^−5^, see Methods). Late adaptive genes were defined as those that were differentially expressed after the 1 hr. high dose following pretreatment (391 genes). In comparison, a much larger set of 1,893 genes was differentially expressed in response to the high dose in the absence of pretreatment.

The overlap of the acute expression response with either the early or late adapted responses was significant (p = 2.1×10^−2^ versus p = 6.8×10^−36^ by hypergeometric test, respectively); nonetheless the overlap with the early response was much less than with the late adapted response (38% versus 60%, see Figure 2C). In addition, 26 genes that would be expected to be increasing in expression based on the acute expression data were decreasing in expression during adaptation, such as genes involved in the response to oxidative stress (GO Biological Process 0006979) (Figure 2B). Other sets of genes were expressed uniquely during early and late adaptation, including ergosterol metabolism, fatty acid synthesis, and zinc homeostasis (GO Biological Processes 0008204, 0006631, 0055069, respectively) (Figure 2B). Unlike the fitness profiling, oxidative stress genes were strongly implicated in the acute expression response (as also found by others; Tables S2 and S3).

### Centrality of Transcription Factors Mga2, Rox1, and Yap1 during Pretreatment Expression

**Figure 4:**
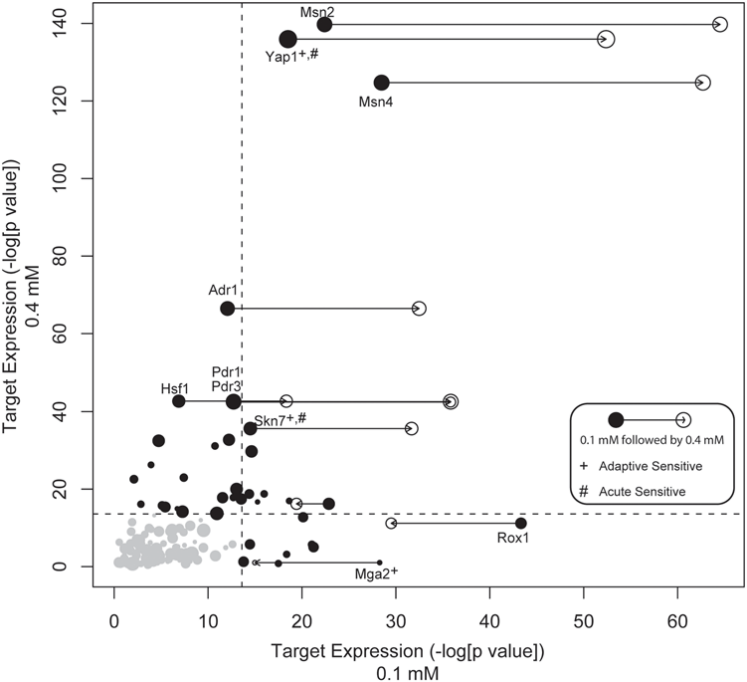
Dynamics of transcription factor target expression in mild and acute conditions. For each transcription factor, we compute a score based on a hypergeometric test representing the significance of increased expression (relative to untreated) of known targets (see Methods) following either pretreatment (0.1 mM H_2_O_2_, x-axis) or acute treatment (0.4 mM H_2_O_2_, y-axis). For those transcription factors with the most significant activity following pre- or acute treatment, the activity following adaptive treatment (0.1 mM followed by 0.4 mM H_2_O_2_) is also displayed on the x-axis with an open circle. The size of each point corresponds to the number of known targets of that transcription factor. The dotted lines indicate a threshold for significance determined by a randomization procedure (see Methods). Although there is significant overlap in the set of expressed genes following mild and acute treatment, examination of specific transcription factors reveals those with unique behavior in each condition. Transcription factors identified in the deletion fitness analysis of the acute and adaptive treatments are indicated with “#” and “+” symbols, respectively.

To map the transcriptional program underlying adaptation, we computed the activity of each yeast transcription factor based on the significance of differential expression among its set of known targets (Figure 4). Lists of targets for each factor were drawn from YeastRACT, a database of literature-curated regulatory interactions [44] (Methods). Application of this method to the acute treatment protocol identified Msn2/4, Yap1, and Skn7 as key factors, all of which had been previously associated with the acute response to oxidative stress. All of these factors were also moderately active during pretreatment and became more so after transitioning to the high dose (Figure 4). Other factors exhibiting this behavior include Adr1, Hsf1, and Pdr1/3.

On the other hand, targets of Mga2 and Rox1 exhibited highly significant activity during pretreatment, but not during the acute response (Figure 4). As Rox1 is a transcriptional repressor, the up-regulation of its targets suggests a decrease in Rox1 activity [45]. While *mga2Δ* was also identified as an adaptive-deficient strain in the high-throughput screen (Figure 2), *rox1Δ* was not. Both of these findings were confirmed with targeted investigations of individual deletion strains (Figure 3). Like Mga2, Rox1 had previously been associated with the hypoxic, not oxidative, stress response [46]. Thus, our analysis appears to classify transcription factors into two categories: early response factors activated by mild doses of oxidant during pretreatment only (Rox1, Mga2), and late damage response factors whose level of activation responds in proportion to treatment dose (Msn2/4, Yap1, Skn7).

### Deletion Studies Confirm the Influence of Mga2, Rox1, and Yap1 on Gene Expression

**Figure 5:**
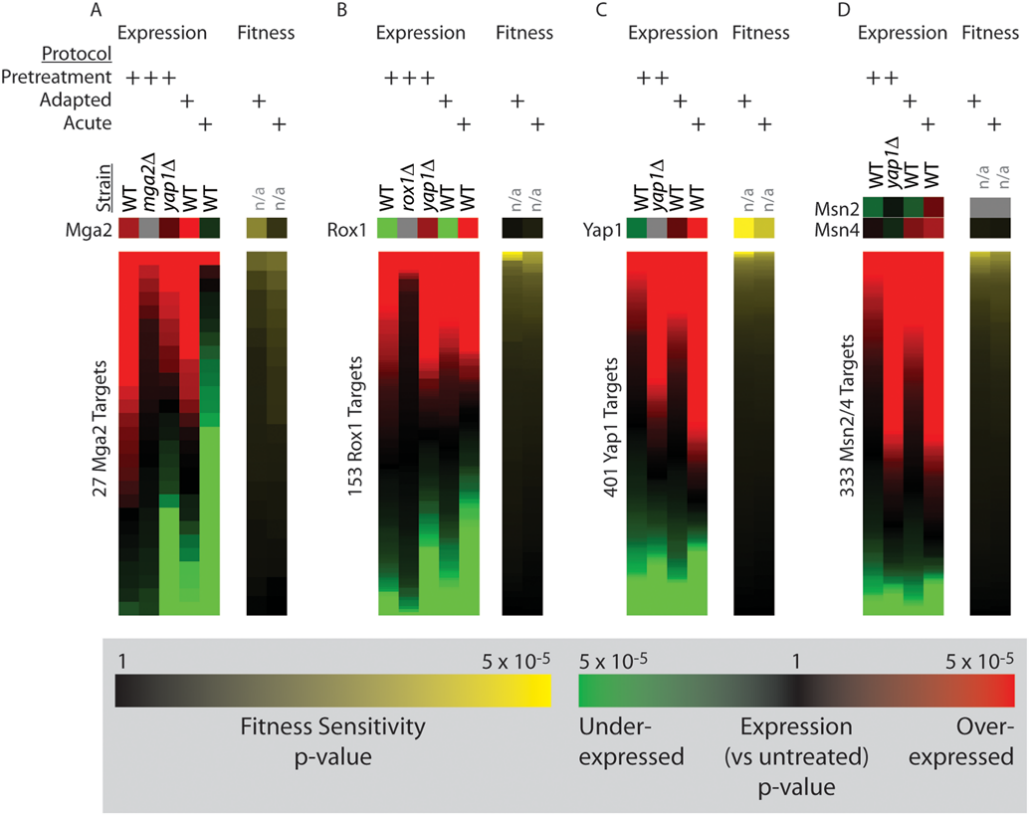
Expression analysis of deletion mutants validates the activation of key transcription factors in response to H_2_O_2_ pretreatment. Panels A–D detail the behavior of the transcription factors Mga2, Rox1, Yap1, and Msn2/4 and their target sets, respectively. Each column represents the expression or fitness values in sorted order for a specific set of genes.

The involvement of Mga2 in early adaptation is supported by its requirement for adaptive growth in the deletion profiling experiments (Figures 2 and 3) and the striking behavior of its targets in the expression profiling experiments (Figure 4). To further confirm the activity of Mga2, pretreatment with hydrogen peroxide was repeated in an *mga2*Δ background and gene expression was profiled versus untreated cells using quadruplicate whole-genome microarrays. In this experiment, the number of up-regulated Mga2 targets was significantly decreased (Figure 5A, p = 1.2×10^−2^ by Fisher's Exact Test), supporting the activation of Mga2 by mild pretreatment with hydrogen peroxide. Moreover, the *MGA2* gene is itself up-regulated following pretreatment and the transition to the high dose (p = 1.4×10^−3^ and 5.3×10^−5^, respectively).

Rox1 (Repressor of Hypoxic Genes) is a repressor under transcriptional control of Hap1 [45]. The decrease in expression of the *ROX1* gene following both the pretreatment and adapted treatment protocols (p = 3.6×10^−11^ and 1.4×10^−7^, respectively) suggests that this repressor is deactivated in the process of adaptation. To confirm this observation we profiled a *rox1*Δ strain and found that the number of Rox1 targets with increased expression following pretreatment falls significantly (p = 0.046 by Fisher's Exact Test) (Figure 5B). However, as we cannot demonstrate a fitness requirement for Rox1, it is unclear whether the expression changes due to de-repression by Rox1 are functionally relevant.

A similar expression analysis suggests that Yap1 is an active regulator during both the pretreatment and high-dose phases of adaptation. To confirm the activity of Yap1 during pretreatment, we profiled the expression response of a *yap1*Δ strain versus *wild type* cells under the pretreatment protocol. This experiment revealed widespread changes in patterns of expression (Figure 5C). The expression responses of Yap1, Rox1, and Msn2/4 targets following mild pretreatment in the *yap1*Δ strain most closely resembled their expression responses in the *wild type* following acute treatment (Figure 5B–D). Thus, it is clear that Yap1 is required for many of the expression changes associated with adaptation.

Interestingly, Mga2, Rox1, and Yap1 targets were not enriched for genes that were required for adaptation in the competitive fitness screen (Figure 5A,B,C; Dataset S2 gives a list of all required targets). In the case of Mga2, not a single target gene was required for adaptation. This suggests significant functional redundancy in the genes targeted by these factors, or that their requirement for adaptation is mediated by targets that are essential for viability and therefore are not included in the deletion strain collection used in the screen for competitive fitness.

### Potential Mechanisms of Mga2 and Rox1 Activation

The mechanisms by which Mga2 and Rox1 might be activated by mild pretreatment with oxidants are unknown, but several lines of evidence suggest they are shared with the hypoxic response. Rox1 is expressed in a heme-dependent manner [47]. While falling heme levels typically signal hypoxic conditions [48], hydrogen peroxide may also reduce heme levels via degradation [49]. Dirmeier *et al.* found that ROS levels transiently increase following exposure to anoxic conditions, suggesting that this could signal the expression of hypoxic genes [50]. They did not believe the activation of hypoxic genes could be replicated with exogenously supplied ROS, based on the H_2_O_2_ expression profiling data of Causton *et al.* [36]. We contradict this earlier hypothesis with the observation of increased expression of hypoxic genes as a result of treatment with H_2_O_2_. The apparent discrepancy may be a result of the higher dose of H_2_O_2_ used by Causton *et al.* [36].

### Potential Mechanisms of Mga2 Action: Ergosterol Metabolism

In response to mild pretreatment with hydrogen peroxide, Mga2 and Rox1 activate targets involved in ergosterol metabolism, zinc homeostasis, and fatty acid metabolism. Ergosterol is a cholesterol-like component of the plasma membrane with diverse effects on its function [51]. Branco *et al.* observed that adaptation is associated with an increase in membrane rigidity, an effect that is abrogated in the ergosterol-deficient *erg3Δ* and *erg6Δ* strains [52]. Thus, a potential mechanism for Mga2's requirement during adaptation is that it promotes an increase in ergosterol which inhibits diffusion of H_2_O_2_ across the plasma membrane. Zinc homeostasis genes may play a similar role, as these genes also influence ergosterol metabolism [53]. Conversely, Tafforeau *et al.* observed a decrease of both squalene synthase (Erg9) activity and ergosterol content during adaptation in *S. pombe*, highlighting the complex relationship between ergosterol and membrane permeability [54].

**Figure 6:**
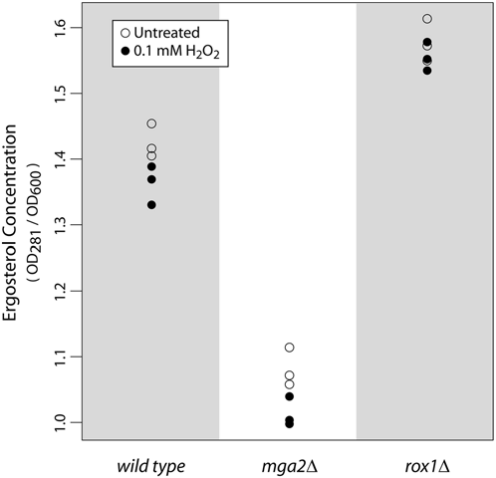
Dynamics of ergosterol following mild treatment with hydrogen peroxide. Following an n-heptane extraction (see Methods), the presence of ergosterol is detected at 281 nm. The ergosterol concentration (relative to the number of cells [OD_600_ value] in the original culture) is reported for *wild type*, *mga2Δ*, and *rox1Δ* strains in three paired trials with and without mild hydrogen peroxide pretreatment.

To elucidate the role of ergosterol biosynthesis in adaptation, we profiled ergosterol concentration in both untreated and adaptive conditions in *wild type*, *mga2Δ*, and *rox1Δ* strains (see Methods). Relative to *wild type*, the basal concentration of ergosterol was significantly lower in the *mga2Δ* strain and slightly higher in the *rox1Δ* strain (Figure 6). This finding agrees with the regulatory roles of Mga2 and Rox1 as an activator and repressor of ergosterol biosynthesis genes, respectively. It also provides some evidence that ergosterol may be a precondition for adaptation to occur, since *mga2Δ* is the only strain tested that had low ergosterol concentration and is also the only one with an adaptation defect (Figure 3). On the other hand, in all strains ergosterol content decreased significantly from untreated to mild pretreated conditions (p = 1.4×10^−2^, 4.1×10^−3^, and 3.1×10^−2^ for *wild type*, *mga2Δ*, *rox1Δ* strains, respectively using a paired t-test). This decrease supports the earlier work of Tafforeau *et al.* [54] but is surprising given it occurs uniformly in all strains, and given that the expression of ergosterol biosynthetic genes increases from untreated to pretreated conditions. One explanation is that expression of ergosterol biosynthetic genes rises in order to compensate for lowered ergosterol levels.

Therefore, we conclude that high ergosterol concentration requires Mga2, supporting a possible role for the influence of Mga2 on ergosterol levels as a precondition of adaptation. However, the change in ergosterol in response to pretreatment does not depend on Mga2 or Rox1, suggesting the involvement of other regulators of ergosterol or of other mechanisms of adaptation that are ergosterol independent.

### Potential Mechanisms of Mga2 Action: Fatty Acids

Two of the most highly expressed genes following pretreatment with hydrogen peroxide were *OLE1* (oleic acid requiring) and *FAS1* (fatty acid synthetase), essential genes required for synthesis of fatty acids. Both genes are direct transcriptional targets of Mga2 (YeastRACT database), suggesting fatty acid pathways as an alternative to ergosterol for the key mechanism of action of Mga2 during adaptation. Although fatty acid pathways could influence the stability and permeability of the plasma membrane [55], these enzymes could also affect the mitochondrial membrane [56], and mutations in *OLE1* have been linked to mitochondrial morphology and inheritance [57].

Because *OLE1* and *FAS1* are essential genes, their specific requirement for adaptation was difficult to assay. However, we found that the high expression of *OLE1* was maintained in a *rox1Δ* background but was greatly reduced in an *mga2Δ* strain (Dataset S3; p = 7.2×10^−3^). Previous work by Matias *et al.* reported decreased expression of *FAS1* mRNA 30 minutes after treatment with 0.15 mM H_2_O_2_ [55]. By 1 hour, no significant differential expression was detected. In comparison, we observed increased expression of *FAS1* one hour after treatment with 0.10 mM H_2_O_2_ and demonstrated that adaptation occurs under these conditions. Thus, *FAS1* has been observed to be both up- and down-regulated during adaptation to H_2_O_2_, albeit at slightly different doses and times. In order to determine the influence of H_2_O_2_ dose and treatment time on *FAS1* expression, we performed RT-PCR profiling of *FAS1* following treatment with both 0.10 mM and 0.15 mM H_2_O_2_. As detailed in Figure S2, we observed an increase in *FAS1* levels following treatment with 0.10 and 0.15 mM H_2_O_2_, although the measurement at 0.15 mM was not significant. This is consistent with both our microarray results and the work of Matias *et al.* Further testing of *FAS1* mRNA levels at 30 minutes following 0.15 mM H_2_O_2_ revealed no significant differential expression (p = 5.6×10^−1^) (Figure S3). Therefore, we have been unable to confirm the previous report of down-regulation of fatty-acid biosynthetic genes during the process of adaptation. Increased expression was also confirmed by RT-PCR for *OLE1* (Figure S2). While the precise adaptation program mediated by Mga2 remains to be elucidated, fatty acid synthesis warrants further study as a possible mechanism.

### Summary and Prospective

**Figure 7:**
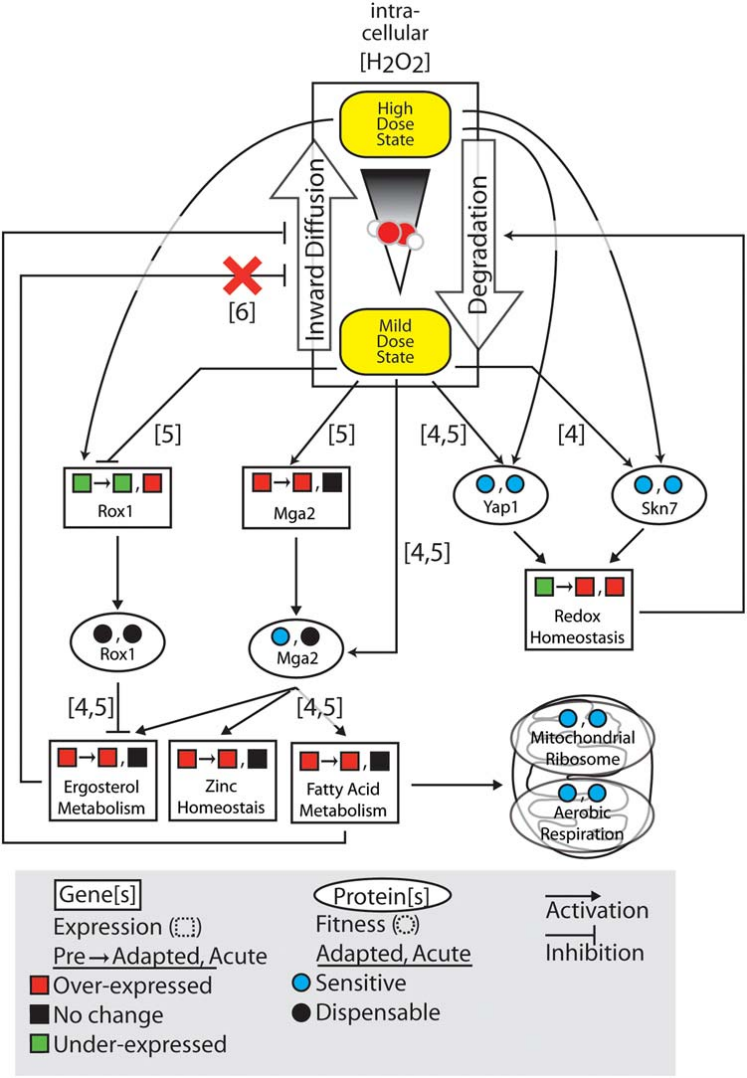
Summary of the adaptive response. Results and hypotheses regarding transcriptional regulators and functional categories identified in this study are summarized. The influence of hydrogen peroxide is determined by its concentration within the cell. In addition to treatment dose, several cellular processes affect the level of H_2_O_2_. In order to enter the cell, hydrogen peroxide must first diffuse across the plasma membrane. Inside the cell, peroxide levels are reduced by degradation into oxygen and water. Squares denote the expression of genes or gene sets (rectangles) following each of the three treatment protocols (pretreatment, adapted, and acute). Conversely, circles denote the sensitivity of the corresponding gene deletion for a particular protein or protein set (oval) in the adapted and acute treatment protocol. Arrows between different objects indicate either an activating (triangular arrowhead) or inhibitory (flat arrowhead) influence. The figure number(s) which provides support for each link are shown in brackets. A red “X” denotes a hypothesis which is refuted by experimental observation.

Figure 7 shows a summary of our findings integrated with previous literature. The expression response during adaptation may be segregated into “early” and “late” phases. “Early” genes respond to pretreatment only and not to the later high dose. Mga2 and Rox1 are likely regulators of the genes involved in the early expression response, with functions in ergosterol biosynthesis, zinc homeostasis, and fatty acid synthesis. Mga2, but not Rox1, is required for maximal adaptive fitness. Conversely, the expression response of “late” genes increases strongly following the high dose of the adaptation protocol. The transcription factors Yap1 and Skn7 have been previously shown to regulate many genes associated with the “late” response, such as those involved in redox homeostasis. In addition, both of these transcription factors are required for adaptation.

One goal for future work is to investigate whether the mechanisms of adaptation identified here also function in higher organisms or in lifespan extension. Of the 156 genes identified in this study as required for the adaptive response, 97 have some homology to higher eukaryotes [58]. In humans, fibroblasts and smooth muscle cells exhibit extended replicative lifespan in response to hypoxic external conditions. This effect requires the generation of ROS inside the cell and the presence of hypoxia inducible factor (HIF). Like Mga2 and Rox1 in *S. cerevisiae*, HIF is a transcription factor that mediates the response to hypoxic conditions, although it is not orthologous to either protein [59],[60]. Further work will be required to see if HIF can be activated not only by hypoxia but also by caloric restriction.

In conclusion, we have completed the first genome-wide scan for genes required for the adaptive response to oxidative stress. By integrating these data with results from expression profiling, we have identified pathways with novel involvement in the response to oxidative stress, including the hypoxic response factor Mga2. The activation of Mga2 under adaptive conditions provides additional information about the sensing mechanism of the hypoxic response, given that we have demonstrated this response can be initiated by exogenous oxidative stress. Future studies can interrogate the manner in which the homologs of these genes are necessary for adaptation in higher organisms and explore their role in aging and disease.

## Methods

### Determination of Treatment Protocols

The high dose of 0.4 mM H_2_O_2_ was selected to be comparable to other previous expression studies of acute hydrogen peroxide exposure (0.4 mM, 0.24 mM, 0.32 mM, for Causton, Shapira, Gasch, respectively) [34]–[36]. This dose resulted in a reduction of growth rate by approximately two thirds as measured by OD_600_. The pretreatment dose was selected as the largest dose that did not result in impaired growth or viability. This criteria and the length of pretreatment (45 minutes) were selected in accordance with previous studies of adaptation to oxidative stress [9],[61],[62].

### Sample Growth and Treatment for mRNA Profiling

We profiled the response to three hydrogen peroxide treatment protocols (pretreatment, adapted, and acute) over a series of microarray experiments. Each series consisted of four biological replicates. For each replicate in the acute treatment protocol, a single colony of BY4741 (ATCC, Manassas, Virginia, USA) was used to inoculate 10 mL of YPD media. Following overnight growth at 30°C, this culture was resuspended in 100 mL of YPD media at an OD_600_ of 0.1 and placed in an orbital shaker at 30°C. At OD_600_ = 0.6 cells were split into two 50 mL portions. In the acute treatment protocol growth continued for 45 minutes, at which point a high dose of hydrogen peroxide (final concentration in media: 0.4 mM H_2_O_2_) was administered to one member of the pair (with the other receiving a sham treatment of 100 mM phosphate buffer). Treatment continued for 1 hour at which point cells were harvested by centrifugation at 3000 rpm for 5 min. Pellets were immediately frozen in liquid nitrogen and stored at −80°C. The pretreatment protocol was identical except for the final concentration of hydrogen peroxide (0.1 mM). For the adapted treatment, a pretreatment dose of hydrogen peroxide (0.1 mM) and corresponding sham treatment were administered directly after splitting the culture, but otherwise the treatment was identical to the acute protocol.

### Strain Construction

All single deletions were obtained from the complete yeast deletion collection in the BY4741 background (ATCC, #2013888) and verified by PCR (http://www.sequence.stanford.edu/group/yeast_deletion_project/single_tube_protocol.html).

### mRNA Expression Analysis

RNA from each sample was isolated via phenol extraction followed by mRNA purification [Poly(A)Purist, Ambion, Catalog # 1916]. Purified mRNA from the control experiments was labeled with dUTP incorporating either Cy3 or Cy5 dye (CyScribe First-Strand cDNA labeling kit, Amersham Biosciences). Cy3 and Cy5 labelings were alternated between replicates to create a balanced design. Complementary labelings (Cy3 versus Cy5) were hybridized to Agilent expression arrays (Catalog # G4140B).

Arrays were scanned using a GenePix 4000A or PerkinElmer Scanarray Lite microarray scanner and quantified with the GenePix 6.0 software package. Data from each array were subjected to background and quantile normalization [63]. Intensity values are available at the GEO database (www.ncbi.nlm.nih.gov/geo/) under the accession number GSE12602. The VERA software package was used with dye bias correction [64] to assign a significance value *λ* of differential expression to each gene. In a negative control experiment (quadruplicate untreated vs. untreated arrays), the distribution of significance values *λ* over all genes was fit parametrically as 1.7 * χ^2^_1_, where χ^2^_1_ is the chi square distribution with one degree of freedom. This null distribution was used for assignment of p-values.

### RT-PCR Expression Analysis

RNA from each sample was isolated by TRIzol extraction (Invitrogen, Catalog # 15596-026) [65]. The purified RNA samples were then used as template for first-strand cDNA synthesis (SuperScript III First-Strand Synthesis for qRT-PCR, Invitrogen, Catalog # 11752-050). For each sample, an RT-PCR reaction was performed with both a gene-specific pair of primers as well as primers targeted to *ACT1*. Sequences for primer pairs are available in Table S4. Each reaction was monitored in triplicate on a 96-well real-time PCR detection system (BIO-RAD MyIQ). For each reaction, this system reports a C_t_ value representing the number of PCR cycles required to exceed a particular fluorescence threshold. The average C_t_ value was calculated across technical replicates for both gene-specific and *ACT1* primer pairs. The mRNA level (reported as the log_2_ ratio relative to the concentration of *ACT1* mRNA) was determined by subtracting the average gene-specific C_t_ value from the average *ACT1* C_t_ value.

### Sample Growth and Treatment for Haploid Deletion Fitness Profiling Experiments

A pool of the 4,831 viable haploid deletion strains was created from individual collections kept in glycerol stock and divided into 1 mL aliquots stored at −80°C. Two separate types of treatment protocols (acute and adapted) were studied consisting of four and six replicate arrays, respectively. For each replicate, a single aliquot of pooled deletion strains was diluted in 15 mL YPD media and grown in a rotating wheel at 30°C to OD_600_ = 0.6. The sample was then split into two 6.5 mL portions. In the adapted treatment protocol, one member of the paired samples was immediately treated with a mild dose of oxidant (final concentration in media: 0.1 mM) and the other received a sham treatment. After 45 minutes of continued growth at 30°C, a high dose was administered (final concentration in media: 0.4 mM) to both samples. After 1 hour of treatment, the cells were harvested by centrifugation at 3000 rpm for 5 min and resuspended in 50 mL of YPD media. After 5 hours of growth, the cells were once again harvested by centrifugation and the pellets were immediately frozen in liquid nitrogen and stored at 80°C. The acute treatment protocol was identical, except that no sample was treated with a mild pretreatment dose and only one member of the sample pair was treated with the high dose.

### Deletion Fitness Analysis

Genomic DNA was extracted from cell pellets using a glass bead preparation [66]. Subsequent DNA labeling, hybridization, and microarray design followed the protocol of Yuan *et al.* [67]. Briefly, asymmetric PCR was used to amplify unique tag sequences in the genomic DNA of the deletion strains. In each PCR reaction, 1 µg of gDNA was used for labeling. Arrays were scanned and quantified in the same manner as the arrays prepared for the expression profiling experiments. Array intensity values are available in the GEO database (www.ncbi.nlm.nih.gov/geo/) under the accession number GSE12733.

The *hoptag* package (implemented in R) was used to analyze the intensity data from the scanned arrays. Briefly, median and loess correction were performed on the intensity distributions [67], after which each deletion strain was assigned an UPTAG ratio and a DNTAG ratio for each array. The logs of these ratios were averaged to derive one measurement per gene per array. Across multiple arrays measuring the same treatment protocol comparison (acute vs. untreated or acute vs. adapted), the distribution of log ratio values was quantile normalized [63].To determine an acute fitness value, we assumed that the signal intensity for a given gene deletion strain is:
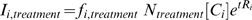
where *I_i,treatment_* and *f_i,treatment_* are the observed signal intensity and viability of gene deletion strain *i* subject to the designated *treatment* protocol, [C*_i_*] and *R_i_* are the initial concentration and growth rate, respectively, of the deletion strain *i*, and *t* is time. *N_treatment_* is a constant factor applied to all intensities from the same treatment representing the shared effect of normalization procedures. For each gene deletion strain *i*, the log ratio of the acute and untreated signal intensities is therefore:
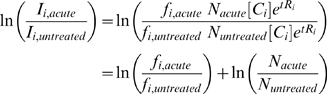
Thus, the log ratio is proportional to the acute fitness metric as defined in Figure 1. Since each intensity distribution was normalized to share the same median, the distribution of log ratios was centered on zero. In order to indentify genes that deviate significantly from this expected value, we performed a one sample *t*-test testing the difference of the mean against zero. This test was regularized to share the estimate of variance among all genes.

Similarly, the log ratio obtained from the direct comparison of the acute and adapted samples was centered on zero and proportional to the log ratio of the viabilities, 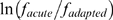. Furthermore, due to median normalization of the intensity distributions, the scales of both log ratio distributions were approximately equal. Thus, for most genes without a defect in adaptive fitness, the log ratio 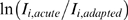 was strongly correlated to the log ratio, 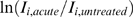. A gene with a large difference between the values 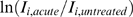 and 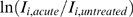 indicated a deviation from the average adaptive fitness measure. A two-sample regularized t-test comparing the log ratios determined from each direct comparison was used to identify such cases. For both adaptive and acute fitness measures, the threshold for significant p-value was set at 5.0×10^−3^.

### Validation of Sensitive Targets

To verify that the identified sensitive genes are meaningful, the sensitivities of specific gene deletions were verified in small-scale experiments. In these, a colony of a specific deletion strain of interest was incubated in YPD overnight. Following dilution to OD_600_ 0.1 in 30 mL YPD media, the culture was grown to OD_600_ 0.6 and split into three aliquots. Each aliquot was treated according to one treatment protocol (untreated, adapted, or acute). Following ten-fold dilution in YPD, growth was monitored in a 96 well optical density plate reader in 12-fold replicate. Examples of recovery following treatment for individual biological replicates are available for *wild type*, *yap1Δ*, *and mga2Δ* in Figures S1, S4, and S5, respectively. For each treatment protocol, the average time required to recover to a particular OD_600_ threshold was determined (Figure 1B). In Figure 3, the specific value of this OD_600_ threshold is varied between 0.3 and 0.95 to illustrate that the substance of the results is not dependent on the selection of any particular value for the threshold. We calculate adaptive fitness as the difference in viability (*f*) between the adaptive and acute treatments relative to the difference between untreated and acute.
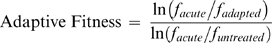
For each treatment protocol, the formula for exponential growth relates the recovery time (*t_treatment_*) to the fractional reduction in viability associated with that treatment (*f_treatment_*), whre *C_threshold_* is the threshold concentration, *C_initial_* is the concentration before treatment, and *r_strain_* is the growth rate of the strain.
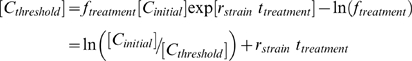
The following derivation illustrates how we can use this information to express the adaptive fitness measure in terms of recovery time,
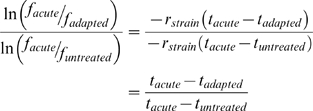
An unpaired t-test was used to determine the significance of the difference from results obtained when applying the same procedure to *wild type* (BY4741) colonies.

### Determination of Ergosterol Concentration

The determination of ergosterol was adapted from Arthington-Skaggs *et al.* [68]. Following overnight incubation, a culture was grown in YPD to OD_600_ 0.6 and split into two aliquots of 50 mL. One of the aliquots was treated with 0.1 mM H_2_O_2_ for 1 hour, after which the OD_600_ of each aliquot was measured. Each aliquot was pelleted and washed once with water. The cleaned pellet was incubated for 1 hour at 85°C with 3 mL 25% alcoholic KOH. After cooling for 15 minutes, 1 mL water and 3 mL n-heptane were added and the mixture was vortexed for 3 minutes. The n-heptane layer was extracted and the presence of ergosterol was detected via absorbance at OD_281_. The ergosterol concentration for each aliquot of the paired trial was reported as the ratio of OD_600_/OD_281_.

### Enrichment Analysis of Gene Sets

We investigated the significance of enrichment for functional classes among both differentially expressed and sensitive genes. Functional classes were defined in one of two ways: (1) classes of genes with common annotation in the Gene Ontology (GO) hierarchy [69]) or (2) classes of genes targeted by the same transcription factor as recorded in the YeastRACT online database [44]. In this database, the list of targets for each factor is compiled from literature sources where each regulatory interaction is backed with experimental evidence. To prevent the identification of redundant or overly general gene ontology categories, we limited the GO analysis to those categories that contained between 5 and 100 genes. Similarly, the YeastRACT database contained several transcription factors with an excessive number of annotated targets (Yap1 alone was annotated with over 1,500). To reduce the incidence of false positives, those studies which contributed over 100 targets for a given factor were discarded (on a per factor basis). While this may eliminate some true interactions, the goal is to generate a smaller set of high-confidence interactions which may be used to accurately assess the activity of given transcription factor. The final set of targets for each transcription factor is available as Dataset S4. A hypergeometric test was used to assess the enrichment of each gene set in the lists of differentially expressed or sensitive genes.

Since the true number of differentially expressed or sensitive genes was unknown and poorly defined, we varied the cutoff for significance between 100 and 500 genes. The minimal p-value for each gene set was returned, and the activity/sensitivity of each gene set was reported as the negative log of this minimal p-value. Since the corresponding p-value was no longer strictly accurate as a consequence of multiple hypothesis testing, significance was assessed by repeated randomization trials in which the order of genes was shuffled. Every gene set was tested and the maximum significance value was retained in each trial. Only those gene sets which exceeded the 95^th^ quantile in this set were determined to be significant.

## Supporting Information

Figure S1 Growth of *wild type* following three different treatment protocols. Following treatment with either the acute, adapted, or untreated protocols, *wild type* cultures are diluted 10-fold in YPD. Recovery is monitored with a 96-well OD_600_ plate reader. Each line represents the average of 12 replicates.
(0.15 MB PDF)

Figure S2 RT-PCR profiling of *OLE1* and *FAS1* following H_2_O_2_ treatment. mRNA levels of both *FAS1* and *OLE1* are profiled 60 minutes following treatment with either 0.10 mM or 0.15 mM H_2_O_2_. Levels are normalized to *ACT1* and reported as a log ratio relative to untreated.
(0.06 MB PDF)

Figure S3 RT-PCR profiling of *FAS1* mRNA levels at different time points. The level of *FAS1* mRNA is profiled at 30 and 60 minutes following treatment with 0.15 mM H_2_O_2_ with RT-PCR. mRNA levels are normalized relative to *ACT1* and reported as a log_2_ ratio relative to an untreated sample. Reported p-values are determined with a one-sample t-test testing the difference from a true mean of zero.
(0.04 MB PDF)

Figure S4 Growth of *yap1Δ* following three different treatment protocols (adapted, acute, untreated). Following treatment with either the acute, adapted, or untreated protocols, *yap1Δ* cultures are diluted 10-fold in YPD. Recovery is monitored with a 96-well OD_600_ plate reader. Each line represents the average of 12 replicates.
(0.15 MB PDF)

Figure S5 Growth of *mga2Δ* following three different treatment protocols (adapted, acute, untreated). Following treatment with either the acute, adapted, or untreated protocols, *mga2Δ* cultures are diluted 10-fold in YPD. Recovery is monitored with a 96-well OD_600_ plate reader. Each line represents the average of 12 replicates.
(0.16 MB PDF)

Table S1 Sensitive gene ontology categories following acute hydrogen peroxide stress. For our study and the study of Thorpe *et al.*, we determined those gene ontology categories which were enriched for sensitive gene deletions. Here we report all categories which exceed the threshold for significance.
(0.16 MB PDF)

Table S2 Up-regulated transcription factor target sets following acute hydrogen peroxide stress. For our and previous comparable studies (Gasch 2000, Causton 2001, Shapira 2004), the set of known targets for each transcription factor was ranked based on enrichment for genes with increased expression in response to acute hydrogen peroxide stress. Here, we report the top nine sets of transcription factor targets. To facilitate comparison, frequently occurring items are high-lighted in a consistent manner.
(0.13 MB PDF)

Table S3 Up- and down-regulated gene ontology categories following acute hydrogen peroxide stress. For our and previous comparable studies (Gasch 2000, Causton 2001, Shapira 2004), a pruned set of functional categories was ranked based on enrichment for genes with increased and decreased expression in response to acute hydrogen peroxide stress. In each case, we report the top five categories. To facilitate comparison, frequently occurring categories are high-lighted in a consistent manner.
(0.62 MB PDF)

Table S4 Primer sequences for RT-PCR profiling of gene expression.
(0.05 MB PDF)

Dataset S1 Fitness Table: P-values for acute and adaptive screens conducted in this study.
(0.20 MB TXT)

Dataset S2 Enrichment Summary: Differentially expressed or sensitive members of each significantly over-represented condition or transcription factor target set mentioned in the study.
(0.04 MB TXT)

Dataset S3 Expression Table: Log ratios and p-values for all micro-array expression profiling experiments conducted in this study.
(1.05 MB TXT)

Dataset S4 TFs Table: Table containing all of the transcription factor target sets used in this study.
(0.11 MB TXT)

## References

[1] Lusis AJ (2000). Atherosclerosis.. Nature.

[2] Jenner P (2003). Oxidative stress in Parkinson's disease.. Ann Neurol.

[3] Markesbery WR (1997). Oxidative stress hypothesis in Alzheimer's disease.. Free Radic Biol Med.

[4] Christen Y (2000). Oxidative stress and Alzheimer disease.. Am J Clin Nutr.

[5] Weindruch R, Naylor PH, Goldstein AL, Walford RL (1988). Influences of aging and dietary restriction on serum thymosin alpha 1 levels in mice.. J Gerontol.

[6] Harman D (1956). Aging: a theory based on free radical and radiation chemistry.. J Gerontol.

[7] Lin SJ, Kaeberlein M, Andalis AA, Sturtz LA, Defossez PA (2002). Calorie restriction extends Saccharomyces cerevisiae lifespan by increasing respiration.. Nature.

[8] Schulz TJ, Zarse K, Voigt A, Urban N, Birringer M (2007). Glucose restriction extends Caenorhabditis elegans life span by inducing mitochondrial respiration and increasing oxidative stress.. Cell Metab.

[9] Jamieson DJ (1992). Saccharomyces cerevisiae has distinct adaptive responses to both hydrogen peroxide and menadione.. J Bacteriol.

[10] Kim DK, Cho ES, Lee BR, Um HD (2001). NF-kappa B mediates the adaptation of human U937 cells to hydrogen peroxide.. Free Radic Biol Med.

[11] Lee BR, Um HD (1999). Hydrogen peroxide suppresses U937 cell death by two different mechanisms depending on its concentration.. Exp Cell Res.

[12] Wiese AG, Pacifici RE, Davies KJ (1995). Transient adaptation of oxidative stress in mammalian cells.. Arch Biochem Biophys.

[13] Masoro EJ (2000). Caloric restriction and aging: an update.. Exp Gerontol.

[14] Masoro EJ (2005). Overview of caloric restriction and ageing.. Mech Ageing Dev.

[15] Costa V, Moradas-Ferreira P (2001). Oxidative stress and signal transduction in Saccharomyces cerevisiae: insights into ageing, apoptosis and diseases.. Mol Aspects Med.

[16] Meister A (1988). Glutathione metabolism and its selective modification.. J Biol Chem.

[17] Muller EG (1992). Thioredoxin genes in Saccharomyces cerevisiae: map positions of TRX1 and TRX2.. Yeast.

[18] Gan ZR (1991). Yeast thioredoxin genes.. J Biol Chem.

[19] Alvarez-Peral FJ, Zaragoza O, Pedreno Y, Arguelles JC (2002). Protective role of trehalose during severe oxidative stress caused by hydrogen peroxide and the adaptive oxidative stress response in Candida albicans.. Microbiology.

[20] Benaroudj N, Lee DH, Goldberg AL (2001). Trehalose accumulation during cellular stress protects cells and cellular proteins from damage by oxygen radicals.. J Biol Chem.

[21] Petrova VY, Drescher D, Kujumdzieva AV, Schmitt MJ (2004). Dual targeting of yeast catalase A to peroxisomes and mitochondria.. Biochem J.

[22] Hartig A, Ruis H (1986). Nucleotide sequence of the Saccharomyces cerevisiae CTT1 gene and deduced amino-acid sequence of yeast catalase T.. Eur J Biochem.

[23] Bermingham-McDonogh O, Gralla EB, Valentine JS (1988). The copper, zinc-superoxide dismutase gene of Saccharomyces cerevisiae: cloning, sequencing, and biological activity.. Proc Natl Acad Sci U S A.

[24] Ravindranath SD, Fridovich I (1975). Isolation and characterization of a manganese-containing superoxide dismutase from yeast.. J Biol Chem.

[25] Stephen DW, Rivers SL, Jamieson DJ (1995). The role of the YAP1 and YAP2 genes in the regulation of the adaptive oxidative stress responses of Saccharomyces cerevisiae.. Mol Microbiol.

[26] Jackson AL, Loeb LA (2001). The contribution of endogenous sources of DNA damage to the multiple mutations in cancer.. Mutat Res.

[27] Hasan R, Leroy C, Isnard AD, Labarre J, Boy-Marcotte E (2002). The control of the yeast H2O2 response by the Msn2/4 transcription factors.. Mol Microbiol.

[28] Stephen DW, Jamieson DJ (1996). Glutathione is an important antioxidant molecule in the yeast Saccharomyces cerevisiae.. FEMS Microbiol Lett.

[29] Kistler M, Summer KH, Eckardt F (1986). Isolation of glutathione-deficient mutants of the yeast Saccharomyces cerevisiae.. Mutat Res.

[30] Izawa S, Inoue Y, Kimura A (1995). Oxidative stress response in yeast: effect of glutathione on adaptation to hydrogen peroxide stress in Saccharomyces cerevisiae.. FEBS Lett.

[31] Alic N, Felder T, Temple MD, Gloeckner C, Higgins VJ (2004). Genome-wide transcriptional responses to a lipid hydroperoxide: adaptation occurs without induction of oxidant defenses.. Free Radic Biol Med.

[32] Grant CM, MacIver FH, Dawes IW (1997). Mitochondrial function is required for resistance to oxidative stress in the yeast Saccharomyces cerevisiae.. FEBS Lett.

[33] Flattery-O'Brien J, Collinson LP, Dawes IW (1993). Saccharomyces cerevisiae has an inducible response to menadione which differs from that to hydrogen peroxide.. J Gen Microbiol.

[34] Gasch AP, Spellman PT, Kao CM, Carmel-Harel O, Eisen MB (2000). Genomic expression programs in the response of yeast cells to environmental changes.. Mol Biol Cell.

[35] Shapira M, Segal E, Botstein D (2004). Disruption of yeast forkhead-associated cell cycle transcription by oxidative stress.. Mol Biol Cell.

[36] Causton HC, Ren B, Koh SS, Harbison CT, Kanin E (2001). Remodeling of yeast genome expression in response to environmental changes.. Mol Biol Cell.

[37] Thorpe GW, Fong CS, Alic N, Higgins VJ, Dawes IW (2004). Cells have distinct mechanisms to maintain protection against different reactive oxygen species: oxidative-stress-response genes.. Proc Natl Acad Sci U S A.

[38] Ng CH, Tan SX, Perrone GG, Thorpe GW, Higgins VJ (2008). Adaptation to hydrogen peroxide in Saccharomyces cerevisiae: the role of NADPH-generating systems and the SKN7 transcription factor.. Free Radic Biol Med.

[39] Shoemaker DD, Lashkari DA, Morris D, Mittmann M, Davis RW (1996). Quantitative phenotypic analysis of yeast deletion mutants using a highly parallel molecular bar-coding strategy.. Nat Genet.

[40] Winzeler EA, Shoemaker DD, Astromoff A, Liang H, Anderson K (1999). Functional characterization of the S. cerevisiae genome by gene deletion and parallel analysis.. Science.

[41] Raitt DC, Johnson AL, Erkine AM, Makino K, Morgan B (2000). The Skn7 response regulator of Saccharomyces cerevisiae interacts with Hsf1 in vivo and is required for the induction of heat shock genes by oxidative stress.. Mol Biol Cell.

[42] Temple MD, Perrone GG, Dawes IW (2005). Complex cellular responses to reactive oxygen species.. Trends Cell Biol.

[43] Chellappa R, Kandasamy P, Oh CS, Jiang Y, Vemula M (2001). The membrane proteins, Spt23p and Mga2p, play distinct roles in the activation of Saccharomyces cerevisiae OLE1 gene expression. Fatty acid-mediated regulation of Mga2p activity is independent of its proteolytic processing into a soluble transcription activator.. J Biol Chem.

[44] Teixeira MC, Monteiro P, Jain P, Tenreiro S, Fernandes AR (2006). The YEASTRACT database: a tool for the analysis of transcription regulatory associations in Saccharomyces cerevisiae.. Nucleic Acids Res.

[45] Ter Linde JJ, Steensma HY (2002). A microarray-assisted screen for potential Hap1 and Rox1 target genes in Saccharomyces cerevisiae.. Yeast.

[46] Zitomer RS, Lowry CV (1992). Regulation of gene expression by oxygen in Saccharomyces cerevisiae.. Microbiol Rev.

[47] Keng T (1992). HAP1 and ROX1 form a regulatory pathway in the repression of HEM13 transcription in Saccharomyces cerevisiae.. Mol Cell Biol.

[48] Hon T, Dodd A, Dirmeier R, Gorman N, Sinclair PR (2003). A mechanism of oxygen sensing in yeast. Multiple oxygen-responsive steps in the heme biosynthetic pathway affect Hap1 activity.. J Biol Chem.

[49] Nagababu E, Rifkind JM (2004). Heme degradation by reactive oxygen species.. Antioxid Redox Signal.

[50] Dirmeier R, O'Brien KM, Engle M, Dodd A, Spears E (2002). Exposure of yeast cells to anoxia induces transient oxidative stress. Implications for the induction of hypoxic genes.. J Biol Chem.

[51] Daum G, Lees ND, Bard M, Dickson R (1998). Biochemistry, cell biology and molecular biology of lipids of Saccharomyces cerevisiae.. Yeast.

[52] Branco MR, Marinho HS, Cyrne L, Antunes F (2004). Decrease of H2O2 plasma membrane permeability during adaptation to H2O2 in Saccharomyces cerevisiae.. J Biol Chem.

[53] Lyons TJ, Villa NY, Regalla LM, Kupchak BR, Vagstad A (2004). Metalloregulation of yeast membrane steroid receptor homologs.. Proc Natl Acad Sci U S A.

[54] Tafforeau L, Le Blastier S, Bamps S, Dewez M, Vandenhaute J (2006). Repression of ergosterol level during oxidative stress by fission yeast F-box protein Pof14 independently of SCF.. Embo J.

[55] Matias AC, Pedroso N, Teodoro N, Marinho HS, Antunes F (2007). Down-regulation of fatty acid synthase increases the resistance of Saccharomyces cerevisiae cells to H2O2.. Free Radic Biol Med.

[56] Janki RM, Aithal HN, McMurray WC, Tustanoff ER (1974). The effect of altered membrane-lipid composition on enzyme activities of outer and inner mitochondrial membranes of Saccharomyces cerevisiae.. Biochem Biophys Res Commun.

[57] Hermann GJ, Shaw JM (1998). Mitochondrial dynamics in yeast.. Annu Rev Cell Dev Biol.

[58] Tatusov RL, Fedorova ND, Jackson JD, Jacobs AR, Kiryutin B (2003). The COG database: an updated version includes eukaryotes.. BMC Bioinformatics.

[59] Bell EL, Klimova TA, Eisenbart J, Schumacker PT, Chandel NS (2007). Mitochondrial reactive oxygen species trigger hypoxia-inducible factor-dependent extension of the replicative life span during hypoxia.. Mol Cell Biol.

[60] Minamino T, Mitsialis SA, Kourembanas S (2001). Hypoxia extends the life span of vascular smooth muscle cells through telomerase activation.. Mol Cell Biol.

[61] Collinson LP, Dawes IW (1992). Inducibility of the response of yeast cells to peroxide stress.. J Gen Microbiol.

[62] Davies JM, Lowry CV, Davies KJ (1995). Transient adaptation to oxidative stress in yeast.. Arch Biochem Biophys.

[63] Bolstad BM, Irizarry RA, Astrand M, Speed TP (2003). A comparison of normalization methods for high density oligonucleotide array data based on variance and bias.. Bioinformatics.

[64] Kelley R, Feizi H, Ideker T (2007). Correcting for gene-specific dye bias in DNA microarrays using the method of maximum likelihood.. Bioinformatics.

[65] Chomczynski P, Sacchi N (1987). Single-step method of RNA isolation by acid guanidinium thiocyanate-phenol-chloroform extraction.. Anal Biochem.

[66] Kaiser C, Adams A, Cold Spring Harbor Laboratory. (1998). Methods in yeast genetics : a Cold Spring Harbor Laboratory course manual.

[67] Yuan DS, Pan X, Ooi SL, Peyser BD, Spencer FA (2005). Improved microarray methods for profiling the Yeast Knockout strain collection.. Nucleic Acids Res.

[68] Arthington-Skaggs BA, Jradi H, Desai T, Morrison CJ (1999). Quantitation of ergosterol content: novel method for determination of fluconazole susceptibility of Candida albicans.. J Clin Microbiol.

[69] Ashburner M, Ball CA, Blake JA, Botstein D, Butler H (2000). Gene ontology: tool for the unification of biology. The Gene Ontology Consortium.. Nat Genet.

