# Supplementary figures and images for: Genome-Wide Fitness and Expression Profiling Implicate Mga2 in Adaptation to Hydrogen Peroxide
Source: PLoS Genet. 2009 May 29;5(5):e1000488. doi: 10.1371/journal.pgen.1000488 (PMC2676504; doi:10.1371/journal.pgen.1000488)

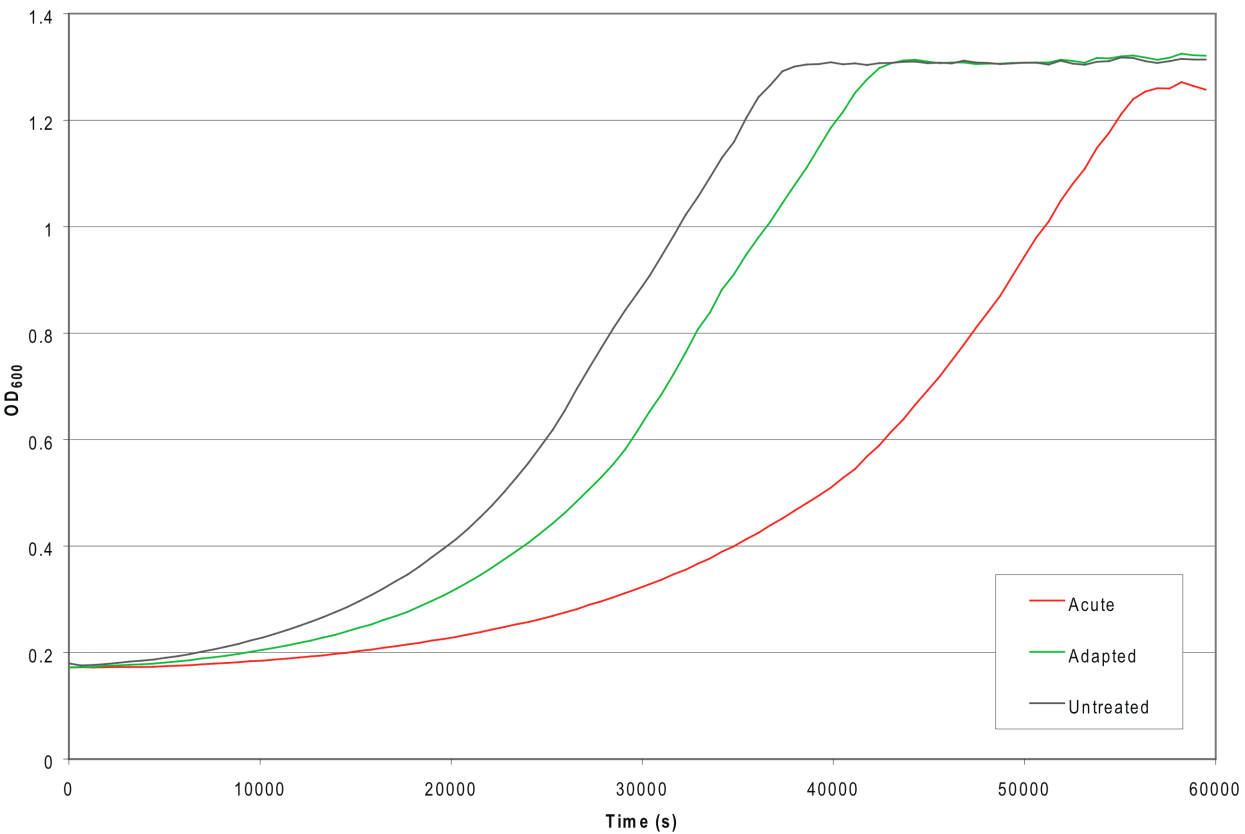

Supplement: Figure S1 — Growth of wild type following three different treatment protocols. Following treatment with either the acute, adapted, or untreated protocols, wild type cultures are diluted 10-fold in YPD. Recovery is monitored with a 96-well OD600 plate reader. Each line represents the average of 12 replicates. (0.15 MB PDF) [file pgen.1000488.s001.pdf]

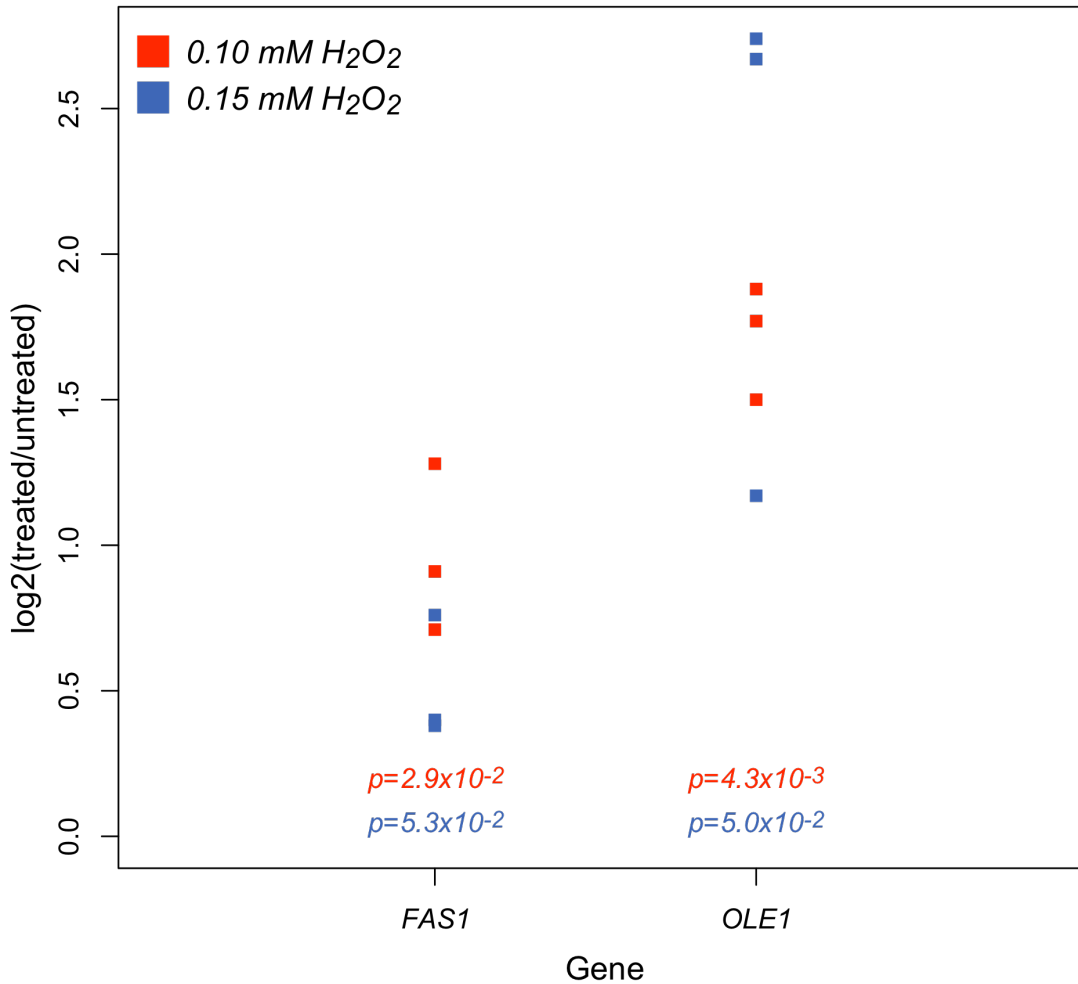

Supplement: Figure S2 — RT-PCR profiling of OLE1 and FAS1 following H2O2 treatment. mRNA levels of both FAS1 and OLE1 are profiled 60 minutes following treatment with either 0.10 mM or 0.15 mM H2O2. Levels are normalized to ACT1 and reported as a log ratio relative to untreated. (0.06 MB PDF) [file pgen.1000488.s002.pdf]

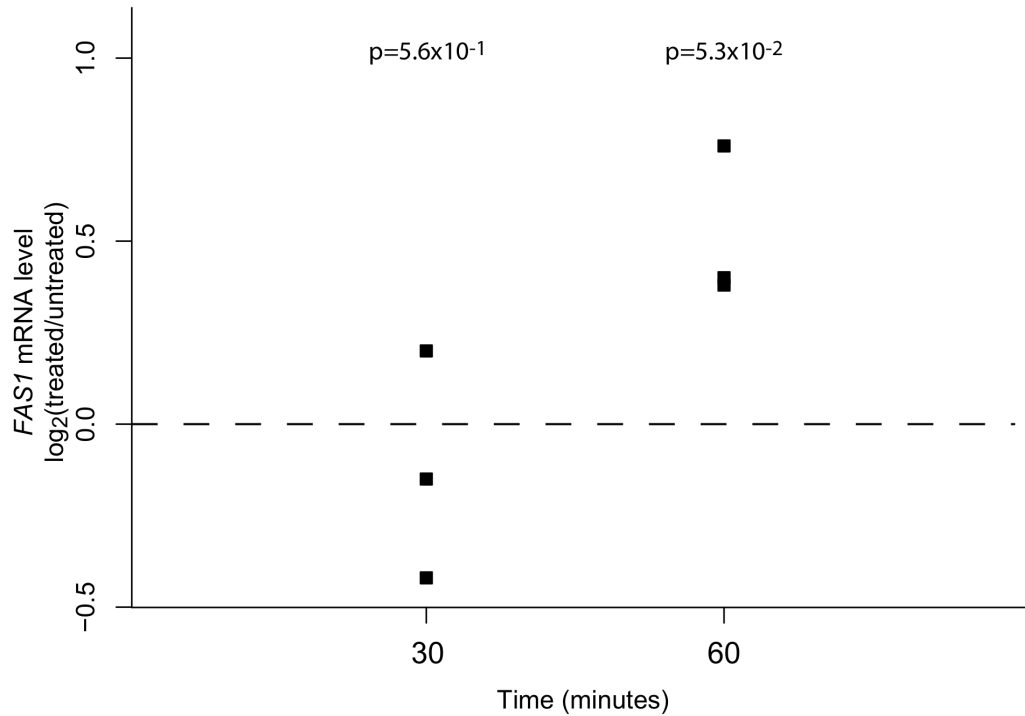

Supplement: Figure S3 — RT-PCR profiling of FAS1 mRNA levels at different time points. The level of FAS1 mRNA is profiled at 30 and 60 minutes following treatment with 0.15 mM H2O2 with RT-PCR. mRNA levels are normalized relative to ACT1 and reported as a log2 ratio relative to an untreated sample. Reported p-values are determined with a one-sample t-test testing the difference from a true mean of zero. (0.04 MB PDF) [file pgen.1000488.s003.pdf]

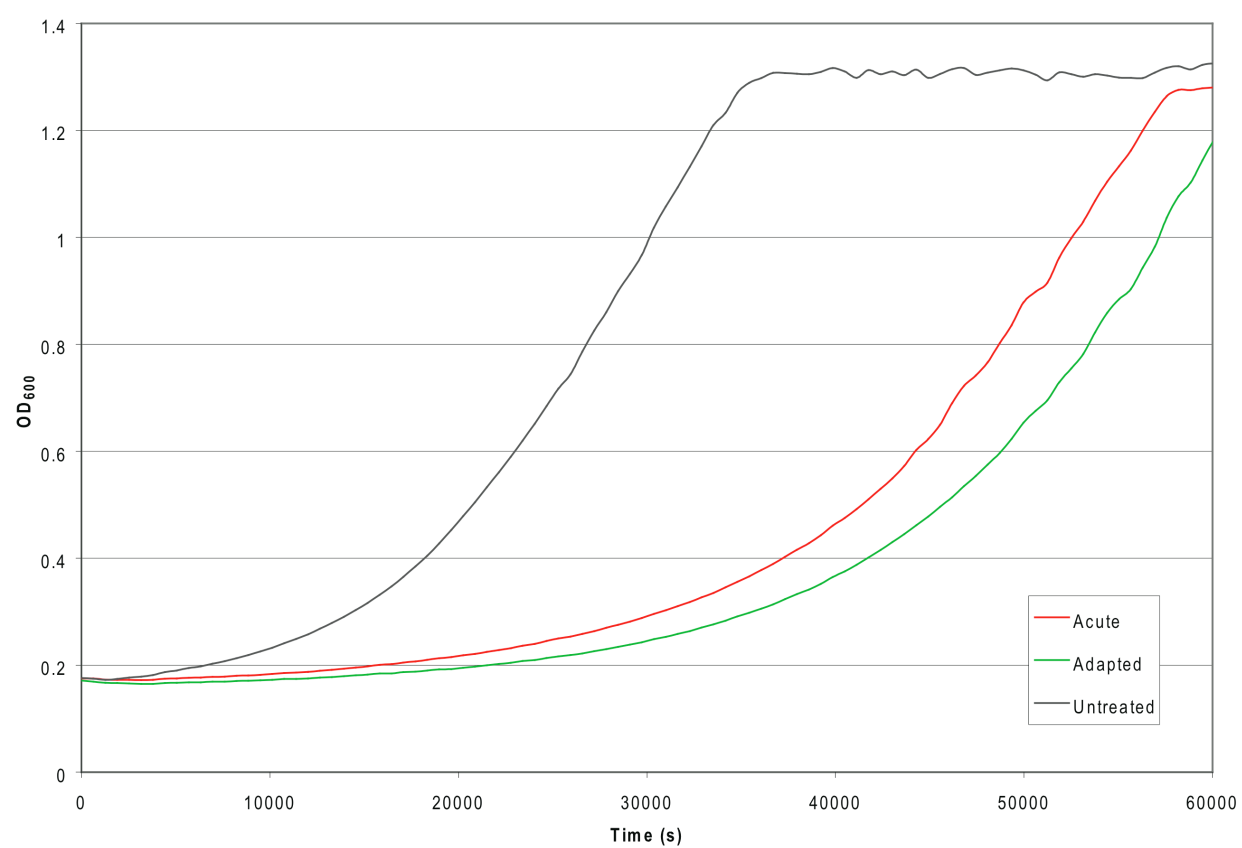

Supplement: Figure S4 — Growth of yap1Δ following three different treatment protocols (adapted, acute, untreated). Following treatment with either the acute, adapted, or untreated protocols, yap1Δ cultures are diluted 10-fold in YPD. Recovery is monitored with a 96-well OD600 plate reader. Each line represents the average of 12 replicates. (0.15 MB PDF) [file pgen.1000488.s004.pdf]

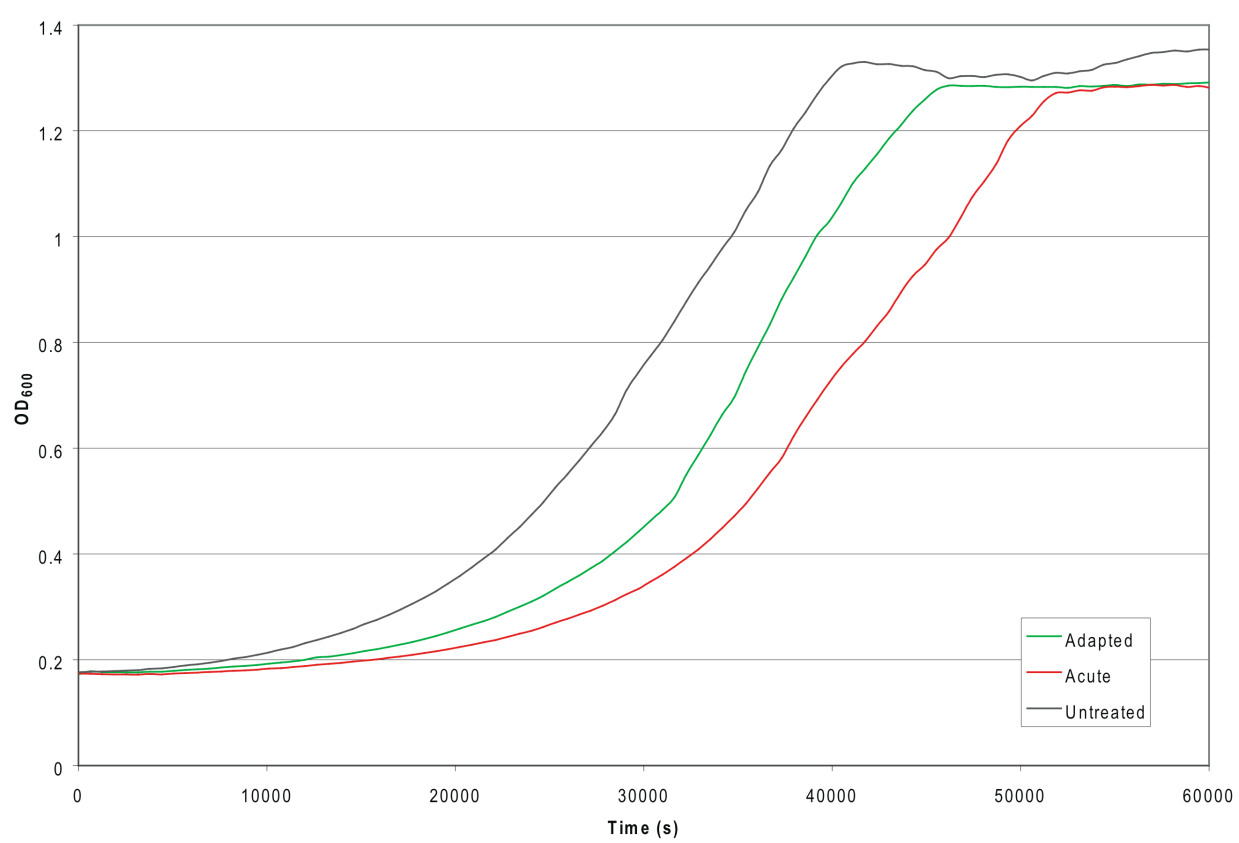

Supplement: Figure S5 — Growth of mga2Δ following three different treatment protocols (adapted, acute, untreated). Following treatment with either the acute, adapted, or untreated protocols, mga2Δ cultures are diluted 10-fold in YPD. Recovery is monitored with a 96-well OD600 plate reader. Each line represents the average of 12 replicates. (0.16 MB PDF) [file pgen.1000488.s005.pdf]
