# Supplementary material for: Genome-Wide Fitness and Expression Profiling Implicate Mga2 in Adaptation to Hydrogen Peroxide
Source: PLoS Genet. 2009 May 29;5(5):e1000488. doi: 10.1371/journal.pgen.1000488 (PMC2676504; doi:10.1371/journal.pgen.1000488)
Supplement: Table S1 — Sensitive gene ontology categories following acute hydrogen peroxide stress. For our study and the study of Thorpe et al., we determined those gene ontology categories which were enriched for sensitive gene deletions. Here we report all categories which exceed the threshold for significance. (0.16 MB PDF) [file pgen.1000488.s006.pdf]

	**Sensitive Gene Ontology Categories**
Thorpe *et al*	mitochondrial ribosome(C)
	aerobic respiration(P)
	mitochondrial protein processing(P)
	nucleoid(C)
	mitochondrial respiratory chain complex III(C)
	tRNA aminoacylation for protein translation(P)
	mitochondrial genome maintenance(P)
Kelley *et al*	mitochondrial ribosome(C)
	aerobic respiration(P)
	mitochondrial transport(P)
	protein processing(P)
	small ribosomal subunit(C)
	double-strand break repair via homologous recombination(P)
	mitochondrial genome maintenance(P)
	endosome membrane(C)
	tRNA aminoacylation for protein translation(P)
	double-strand break repair via single-strand annealing(P)
