# Supplementary material for: Genome-Wide Fitness and Expression Profiling Implicate Mga2 in Adaptation to Hydrogen Peroxide
Source: PLoS Genet. 2009 May 29;5(5):e1000488. doi: 10.1371/journal.pgen.1000488 (PMC2676504; doi:10.1371/journal.pgen.1000488)
Supplement: Table S2 — Up-regulated transcription factor target sets following acute hydrogen peroxide stress. For our and previous comparable studies (Gasch 2000, Causton 2001, Shapira 2004), the set of known targets for each transcription factor was ranked based on enrichment for genes with increased expression in response to acute hydrogen peroxide stress. Here, we report the top nine sets of transcription factor targets. To facilitate comparison, frequently occurring items are high-lighted in a consistent manner. (0.13 MB PDF) [file pgen.1000488.s007.pdf]

Study	Rank	Transcription Factor	Score
Kelley	1	Mns2	139.7
	2	Yap1	136.0
	3	Msn4	124.7
	4	Adr1	66.5
	5	Hsf1	42.7
	6	Pdr1	42.6
	7	Pdr3	42.5
	8	Skn7	35.6
	9	Mig1	32.7
Gasch	1	Msn2	128.4
	2	Msn4	124.6
	3	Yap1	75.3
	4	Pdr1	44.3
	5	Adr1	38.6
	6	Pdr3	38.2
	7	Mig1	26.1
	8	Cad1	25.7
	9	Tos8	22.5
Causton	1	Msn2	136.4
	2	Msn4	132.2
	3	Yap1	80.8
	4	Adr1	53.5
	5	Mig1	39.0
	6	Pdr1	37.7
	7	Cad1	35.6
	8	Pdr3	35.3
	9	Sps18	29.0
Shapira	1	Msn2	89.8
	2	Msn4	89.5
	3	Yap1	61.1
	4	Hsf1	34.1
	5	Pdr3	34.1
	6	Adr1	24.1
	7	Pdr1	23.5
	8	Gis1	22.1
