# Supplementary material for: Genome-Wide Fitness and Expression Profiling Implicate Mga2 in Adaptation to Hydrogen Peroxide
Source: PLoS Genet. 2009 May 29;5(5):e1000488. doi: 10.1371/journal.pgen.1000488 (PMC2676504; doi:10.1371/journal.pgen.1000488)
Supplement: Table S3 — Up- and down-regulated gene ontology categories following acute hydrogen peroxide stress. For our and previous comparable studies (Gasch 2000, Causton 2001, Shapira 2004), a pruned set of functional categories was ranked based on enrichment for genes with increased and decreased expression in response to acute hydrogen peroxide stress. In each case, we report the top five categories. To facilitate comparison, frequently occurring categories are high-lighted in a consistent manner. (0.62 MB PDF) [file pgen.1000488.s008.pdf]

	Gene Ontology Categories	
	Up-regulated	Down-regulated
Kelley	proteasome complex(C)	cytosolic large ribosomal subunit(C)
	response to toxin(P)	small subunit processome(C)
	response to oxidative stress(P)	maturation of SSU-rRNA(P)
	pentose metabolic process(P)	ribosomal large subunit biogenesis and assembly(P)
	carbohydrate catabolic process(P)	cytosolic small ribosomal subunit(C)
	alcohol catabolic process(P)	ribosome assembly(P)
	aldehyde metabolic process(P)	ribonucleoside monophosphate metabolic process(P)
	glycogen metabolic process(P)	nucleobase metabolic process(P)
	mitochondrial intermembrane space(C)	DNA-directed RNA polymerase I complex(C)
Gasch	aldehyde metabolic process(P)	cytosolic large ribosomal subunit(C)
	response to oxidative stress(P)	cytosolic small ribosomal subunit(C)
	response to toxin(P)	ribosomal subunit assembly(P)
	mitochondrial intermembrane space(C)	ribosomal large subunit biogenesis and assembly(P)
	glutathione metabolic process(P)	cell wall(C)
	pentose metabolic process(P)	amine transport(P)
	amino acid derivative catabolic process(P)	carboxylic acid transport(P)
	carbohydrate catabolic process(P)	snRNA metabolic process(P)
	vitamin biosynthetic process(P)	U4/U6 x U5 tri-snRNP complex(C)
Shapira	response to oxidative stress(P)	cytosolic large ribosomal subunit(C)
	aldehyde metabolic process(P)	cytosolic small ribosomal subunit(C)
	trehalose metabolic process(P)	ribosomal large subunit biogenesis and assembly(P)
	response to toxin(P)	ribosome assembly(P)
	siderophore transport(P)	ribosomal small subunit biogenesis and assembly(P)
	vacuolar lumen(C)	small subunit processome(C)
	amino acid derivative catabolic process(P)	ribonucleoside monophosphate biosynthetic process(P)
	proteasome complex(C)	maturation of SSU-rRNA from tricistronic rRNA transcript(P)
	sulfur metabolic process(P)	nucleolar preribosome(C)
Causton	response to oxidative stress(P)	cytosolic large ribosomal subunit(C)
	response to toxin(P)	cytosolic small ribosomal subunit(C)
	pentose metabolic process(P)	small subunit processome(C)
	carbohydrate catabolic process(P)	ribosomal large subunit biogenesis and assembly(P)
	trehalose metabolic process(P)	ribosome assembly(P)
	alcohol catabolic process(P)	maturation of SSU-rRNA(P)
	aldehyde metabolic process(P)	cleavages during rRNA processing(P)
	glutathione metabolic process(P)	maturation of 5.8S rRNA from tricistronic rRNA transcript(P)
