# Supplementary material for: Genome-Wide Fitness and Expression Profiling Implicate Mga2 in Adaptation to Hydrogen Peroxide
Source: PLoS Genet. 2009 May 29;5(5):e1000488. doi: 10.1371/journal.pgen.1000488 (PMC2676504; doi:10.1371/journal.pgen.1000488)
Supplement: Table S4 — Primer sequences for RT-PCR profiling of gene expression. (0.05 MB PDF) [file pgen.1000488.s009.pdf]

Gene	Orientation	Sequence
OLE1	Forward	5'-TGGGACAAACAAACCTTCTTG-3'
	Reverse	5'-AAAGCCTTGGTAGCGTCCTT-3'
FAS1	Forward	5'-TGTTCCTTTCCATTCCACCT-3'
	Reverse	5'-GGCCAATCTAGCAACCTTCA-3'
SEC62	Forward	5'-TCATTGGTGTCGTGTCCATC-3'
	Reverse	5'-ATCCAGAACCCACCAACATC-3'
ACT1	Forward	5'-TATTGCCGAAAGAATGCAAA-3'
	Reverse	5'-GGAAGGTAGTCAAAGAAGCCAAG-3'
